# Ultrafast electron cooling in an expanding ultracold plasma

**DOI:** 10.1038/s41467-020-20815-8

**Published:** 2021-01-26

**Authors:** Tobias Kroker, Mario Großmann, Klaus Sengstock, Markus Drescher, Philipp Wessels-Staarmann, Juliette Simonet

**Affiliations:** 1 The Hamburg Centre for Ultrafast Imaging, Luruper Chaussee 149, 22761 Hamburg, Germany; 2 Center for Optical Quantum Technologies, University of Hamburg, Luruper Chaussee 149, 22761 Hamburg, Germany

**Keywords:** Ultracold gases, Laser-produced plasmas

## Abstract

Plasma dynamics critically depends on density and temperature, thus well-controlled experimental realizations are essential benchmarks for theoretical models. The formation of an ultracold plasma can be triggered by ionizing a tunable number of atoms in a micrometer-sized volume of a ^87^Rb Bose-Einstein condensate (BEC) by a single femtosecond laser pulse. The large density combined with the low temperature of the BEC give rise to an initially strongly coupled plasma in a so far unexplored regime bridging ultracold neutral plasma and ionized nanoclusters. Here, we report on ultrafast cooling of electrons, trapped on orbital trajectories in the long-range Coulomb potential of the dense ionic core, with a cooling rate of 400 K ps^−1^. Furthermore, our experimental setup grants direct access to the electron temperature that relaxes from 5250 K to below 10 K in less than 500 ns.

## Abstract (web summary)

Here the authors report on the creation of ultracold plasma by photoionization of a Bose-Einstein condensate with a femtosecond laser pulse. The experimental setup grants direct access to the electron temperature and reveals ultrafast cooling of electrons in an initially strongly coupled plasma.

## Introduction

Ultrashort laser pulses provide pathways for manipulating and controlling atomic quantum gases on femtosecond timescales. In particular the strong light-field of a femtosecond laser pulse is able to instantaneously ionize a controlled number of atoms in a Bose–Einstein condensate (BEC). Above a critical number of charged particles, the attractive ionic Coulomb potential is large enough to trap a fraction of the photoelectrons, thus forming an ultracold plasma^1^.

**Fig. 1:**
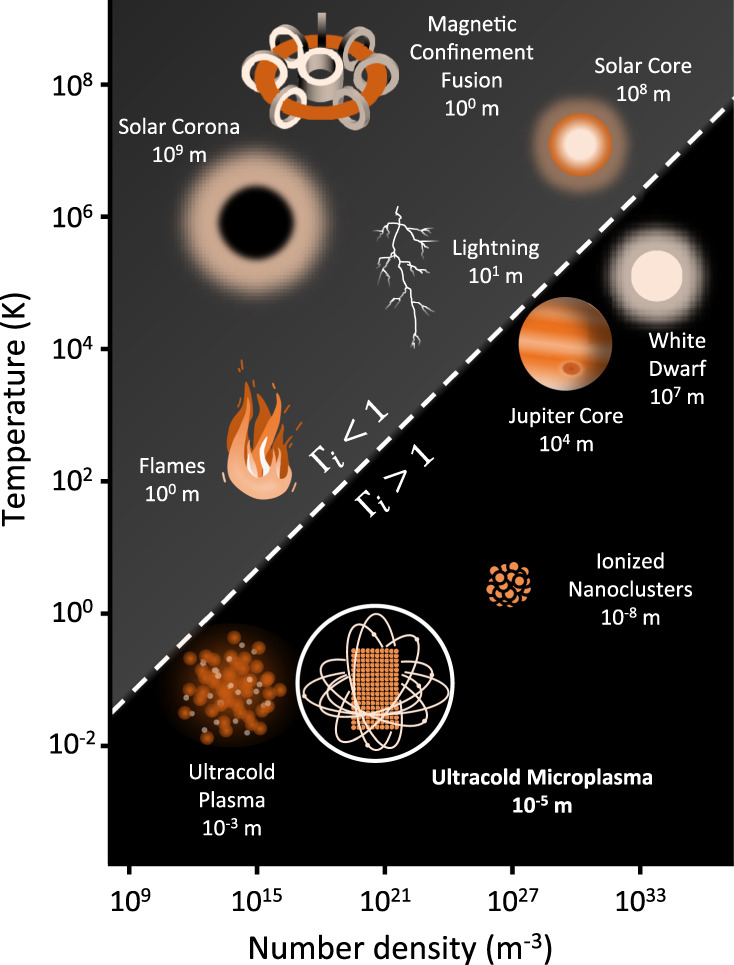
Number density and temperature diagram of plasmas. Plasmas occurring in nature or prepared in the laboratory span several orders of magnitude in size, temperature, and number density. The majority of naturally occurring plasmas are weakly coupled (Γ_i_ < 1); however, the intriguing regime of strongly coupled plasmas (Γ_i_ > 1) is realized in astronomical objects like Jupiter’s core or white dwarfs. The dynamics in this challenging regime can experimentally be approached by ultracold neutral plasmas, ionized nanoclusters, and ultracold microplasmas, where the latter investigated in this work (highlighted by a white circle) bridges the length- and timescales of the former two.

Well-controlled ultracold plasmas in the laboratory provide benchmarks for multi-scale theories and can shed light on extreme conditions present in inertial confinement fusion^2^, the core of Jovian planets and white dwarfs^3^. As depicted in Fig. 1, the plasma density inherited from the BEC surpasses the densities achieved so far in supersonic expansion^4^ and magneto-optical traps (MOTs)^1,5–7^ by orders of magnitude. Strongly coupled plasmas, where the Coulomb energy exceeds the thermal energy, are of particular interest because the charge carriers develop spatial correlations^8^ and self-assembled ordered structures. A recent work^9^ approaches this regime by laser cooling of the ions.

In small clusters ionized by ultrashort laser pulses, strongly coupled plasmas can be realized as well. In such systems, the interplay between interparticle Coulomb energies and molecular bonds is essential to understand energy transfer between electrons and ions^10–12^ (see Fig. 1). Recent experiments have studied charged particle dynamics at solid-state densities in finite-size nanoclusters^13–15^ and observed the emergence of low-energy electrons^16^.

Photoionization of a BEC with a femtosecond laser pulse enables access to an unexplored plasma regime with high charge carrier densities above 10^20^ m^−3^, cold ion temperatures below 40 mK, and hot electron temperatures above 5000 K. Density *ρ* and temperature *T* entirely determine not only the coupling parameter but also the dominating length- and timescales in a plasma. Compared to macroscopic ultracold neutral plasmas (UNPs) at MOT densities, these initial parameters allow for creating a micrometer-sized plasma with large charge imbalance and high plasma frequencies where the Coulomb energies initially exceed the ionic thermal energies by three orders of magnitude. Such an initially strongly coupled microplasma with a few hundred to thousands of particles bridges the dynamics and energy transfer studied in photoionized nanoclusters and UNP. Moreover, this plasma regime allows neglecting three-body-recombination and interatomic binding energies in the theoretical description, which are relevant in UNPs or ionized nanoclusters, respectively. This considerably simplifies theoretical models of the dynamics for benchmark comparisons.

Here, we report on the dynamics of ultracold microplasmas triggered in a ^87^Rb BEC by a femtosecond laser pulse. Our experimental setup grants access to the electronic kinetic energy distribution with meV resolution by combining state-of-the-art techniques of ultrashort laser pulses and ultracold atomic gases. So far, the electron temperature of ultracold plasmas has only been inferred indirectly by comparing the fraction of spilled electrons in an extraction field^17^, the free plasma expansion^18^, or the three-body recombination rate^19^ to theoretical models. We directly observe an electron cooling from 5250 K to below 10 K in <500 ns, which is in excellent agreement with charged particle tracing (CPT) simulations that we have performed in parallel. The small number of particles involved in our microplasma is a key feature that allows for an accurate comparison between experimental results and simulations. In addition, the dynamics investigated here reveals striking effects that cannot be captured by a hydrodynamic description such as the ultrafast electron cooling and the increasing electron coupling parameter approaching unity^6^. Such a laboratory experiment that grants access to additional, microscopic observables, allows testing the validity of macroscopic models, thus leading to a better understanding of similar systems in nature.

## Results

### Ultracold microplasma

**Fig. 2:**
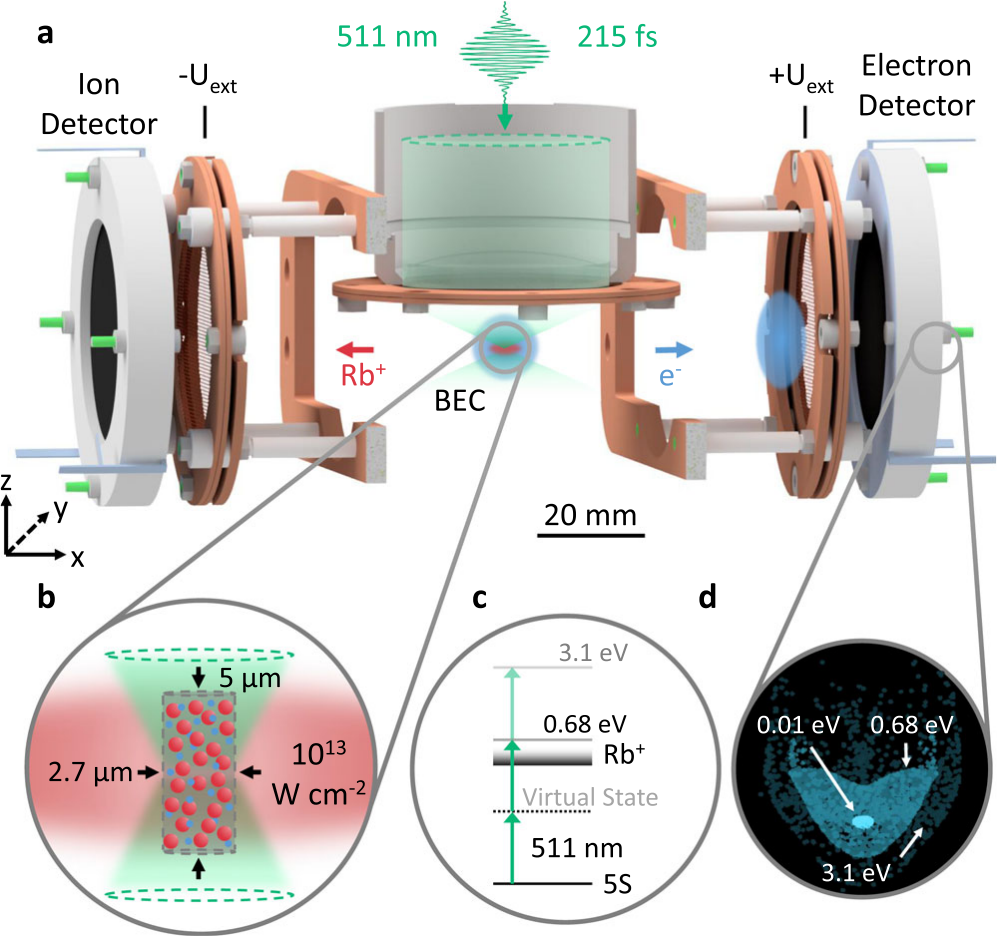
Detection of ionization fragments after ultrafast ionization of a BEC. **a** A single femtosecond laser pulse locally photoionizes a ^87^Rb BEC. The created photoelectrons and ions are separated by an electrical field produced by the two extraction meshes at tunable opposite voltages ±*U*_ext_. Their kinetic energy distribution is converted into a spatial information during the expansion toward a microchannel plate assembly and a phosphor screen, which is imaged by a high-speed camera. **b** At laser peak intensities of 10^13^ W cm^−2^, the femtosecond laser pulse ionizes the majority of atoms within a micrometer-sized cylindrical volume creating a charged particle ensemble immersed in the BEC. **c** At a central wavelength of 511 nm, ^87^Rb is ionized from the 5*S* ground state via nonresonant two- and three-photon processes that correspond to electron excess energies of 0.68 and 3.1 eV, respectively. **d** Simulated electron detector signal after tracing the particle trajectories for three different initial kinetic energies at ±*U*_ext_ = 300 V. The spatial extent on the detector reflects the kinetic energy distribution of the electrons.

We experimentally investigate the dynamics of an ultracold microplasma by combining ultracold quantum gases with the ultrashort timescales of femtosecond laser pulses. As shown in Fig. 2a, a ^87^Rb BEC is locally ionized by a single laser pulse at 511 nm wavelength with a full width at half maximum (FWHM) duration of 215${\,}_{-15}^{+20}$ fs. Whereas earlier photoionization studies in ^87^Rb BECs applied nanosecond laser pulses^20^, here, the pulse duration is significantly shorter than the timescale for the electron dynamics given by the inverse electron plasma frequency

1 $${\omega }_{\mathrm{{p},{e}}}^{-1}=\sqrt{\frac{{m}_{\mathrm{{e}}}{\epsilon }_{0}}{{\rho }_{\mathrm{{e}}}{e}^{2}}}=1.3\,{\text{ps}},$$

where *m*_e_ is the electron mass and *ρ*_e_ is the initial electronic density. Therefore, the initial plasma dynamics is not perturbed by the laser pulse and the creation of the charged particles can be considered as instantaneous.

A high-resolution objective with a numerical aperture of 0.5 focuses the femtosecond laser pulse down to a waist of *w*_0_ ≈ 1 μm leading to peak intensities up to 2 × 10^13^ W cm^−2^ (see “Methods”). The number of ionized atoms can be tuned from a few hundred to *N*_e,i_ ≈ 4000 in a controlled manner via the pulse intensity. At the highest intensity, the ionization probability reaches unity within the center region of a cylindrical volume depicted in Fig. 2b^21^. The radius of 1.35 μm is determined by the laser focus, while the height of 5 μm is limited by the target, thus providing a locally ionized volume within the atomic cloud (see Supplementary Note 1).

As depicted in Fig. 2c, the photoionization of ^87^Rb at 511 nm can be described as a nonresonant two-photon process. The excess energy of 0.68 eV is almost entirely transferred to the lighter photoelectrons. Due to the low initial ionic temperature of *T*_i_ ≈ 33 mK dominated by the photoionization recoil, we attain a remarkably high initial ionic coupling parameter of Γ_i_ = 4800, which compares the Coulomb energy to the thermal energy per particle

2 $${{{\Gamma }}}_{\mathrm{{e},{i}}}=\frac{{e}^{2}}{4\pi {\epsilon }_{0}{a}_{\mathrm{{e},{i}}}{k}_{\mathrm{B}}{T}_{\mathrm{{e},{i}}}}.$$

Here, *T*_e,i_ describes the electron/ion temperature determined by the mean kinetic energy per particle and ${a}_{\mathrm{{e},{i}}}={\left(\frac{3}{4\pi {\rho }_{\mathrm{{e},{i}}}}\right)}^{1/3}$ denotes the Wigner–Seitz radius at the electron/ion density *ρ*_e,i_. As both the interparticle distance and the kinetic energy of the ions increase during plasma evolution, Γ_i_ decreases rapidly (see Supplementary Note 7).

As a key feature, this experimental setup grants access to the atomic density via absorption imaging as well as the energy distribution of the photoionization products (Fig. 2a). A tunable electric field separates electrons and ions and directs them onto opposite imaging microchannel plates (MCP) (see “Methods”). Using CPT simulations, the spatial distribution on the detector can be assigned to an electronic kinetic energy distribution in a quantitative manner (see “Methods”). The simulated detector images for different kinetic energy at ±*U*_ext_ = 300 V are depicted in Fig. 2d: photoelectrons resulting from two-photon ionization (0.68 eV) or from above-threshold ionization (ATI) processes (3.1 eV)^22,23^ can be clearly identified.

### Measurement of electronic temperature

**Fig. 3:**
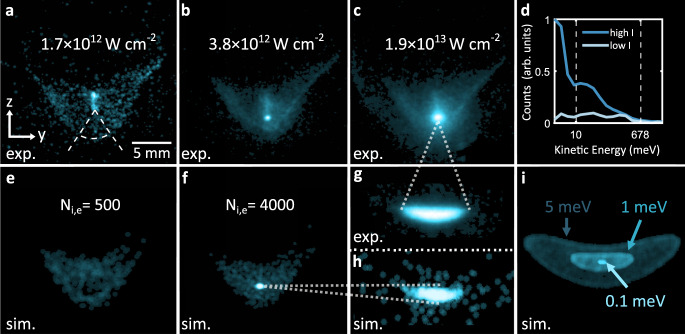
Direct measurements of the electron kinetic energy. **a**–**c** Measured time-integrated electron distributions at ±*U*_ext_ = 300 V and peak intensity of *I*_0_ = 0.17, 0.38 and 1.9 × 10^13^ W cm^−2^ (mean detector image over 18, 23, and 22 realizations). A clear signature for the electrons at the two-photon excess energy is obtained (compare Fig. 2d). As the number of ionized atoms exceeds a critical value, an ultracold plasma is formed signalized here by low kinetic energy electrons (**b**). At the highest intensity, the fraction of slow electrons grows and the signature for three-photon ATI electrons is visible (**c**). **d** Radially averaged spectrum of the recorded electrons at *I*_0_ = 0.17 and 0.38 × 10^13^ W cm^−2^ within the circular sector depicted in **a**. At high intensities, a bright narrow peak appears, corresponding to electrons with kinetic energies below 10 meV. A clear shift of the photoelectrons toward lower energies resulting from the deceleration imposed by the ionic core can be observed. The vertical dashed white lines depict the radii of the electron distributions obtained by trajectory simulations for *E*_kin,e_ = 0.01 and 0.68 eV (see Fig. 2d). **e**, **f** Plasma simulation results for different electron/ion numbers and an initial kinetic energy of 0.68 eV. For *N*_e,i_ = 500 (**e**), we solely observe the two-photon signature while for *N*_e,i_ = 4000 (**f**), slow electrons are produced as consequence of the ultracold plasma dynamics. **g** Measured electron distribution for a peak intensity of *I*_0_ = 1.2 × 10^13^ W cm^−2^ at a lower extraction field of ±*U*_ext_ = 5 V (mean detector image over 14 realizations). **h** Electron distribution obtained for ±*U*_ext_ = 5 V extraction field after plasma simulation with *N*_e,i_ = 4000 for an initial electron kinetic energy of *E*_kin,e_ = 0.68 eV. **i** Trajectory simulation results for initial kinetic energies of *E*_kin,e_ = 0.1, 1, and 5 meV (same spatial scaling as **g** and **h**).

Figure 3a–c shows the averaged electron signals measured for increasing laser pulse peak intensities. For low intensities (a) the dominant structure on the detector is the spatial distribution of the electrons emerging from the nonresonant two-photon ionization process with a kinetic energy of 0.68 eV, corresponding to an initial electron temperature of *T*_e_ ≈ 5250 K, in excellent agreement with the trajectory simulation results (Fig. 2d). For the highest intensity shown (c), a second class of electrons appears stemming from the three-photon ATI (compare to Fig. 2d).

As a central result, in (b) and (c) a narrow peak appears, corresponding to electrons having a very small kinetic energy. At these intensities, the number of photoionized atoms exceeds the critical number of ions N^*^ ≈ 960 required for plasma formation at our excess energies, and a fraction of the photoelectrons is trapped and cooled in the resulting space charge potential generated by the unpaired ions. Experimentally, the threshold intensity depends on the density of the atomic target, which gives a clear evidence of a critical number of ions required for plasma formation (see Supplementary Note 6). This rules out low energetic electrons directly created in the strong-field ionization process as reported at high Keldysh parameters^24^ or speculated in alkali atoms at high intensities^22,25^.

Figure 3d shows radially averaged electron distribution in the depicted circular sector in Fig. 3a. The vertical lines mark the limit of the distributions obtained from trajectory simulations for 0.01 and 0.68 eV depicted in Fig. 2d. At the lowest laser intensity, the kinetic energy distribution is flat up to the energy corresponding to two-photon ionization. At the higher intensity, a large fraction of cold plasma electrons is concentrated below the 1 meV line, which corresponds to a temperature lower than 77 K.

An increasing number of generated ions deepens the space charge potential, which not only enables trapping more electrons in the plasma, but also significantly decelerates the escaping electrons. This can be clearly seen in the averaged spectrum (Fig. 3d) but also as a decrease of the area of the kinetic energy distribution in Fig. 3a–c.

The plasma dynamics is reproduced by CPT plasma simulations including the mutual Coulomb-interactions between all the charged particles (see “Methods”). Figure 3e, f showio/ion numbers of *N*_e,i_ = 500 (e) and 4000 (f) with an initial electron energy of 0.68 eV, which are in excellent agreement with the measured kinetic energy distributions in Fig. 3a, b. In the simulations, slow plasma electrons emerge only above the critical charge carrier density required for plasma formation.

The extraction field sets the expansion time towards the detectors and, thus, the velocity resolution can be tuned from 10 meV at ±*U*_ext_ = 300 V to the 1 meV level at ±*U*_ext_ = 5 V (corresponding to static electric fields of 162 and 4.6 V m^−1^ in the center, respectively). Figure 3g shows the spatial extent of the low-energy plasma electrons as characteristic elliptical structure for an extraction field of 5 V. The trajectory simulation results for different initial energies are depicted in Fig. 3i. Comparison of Fig. 3g, i yields a measured kinetic energy of the plasma electrons of ~1 meV, which corresponds to a final electron temperature below 10 K. Figure 3h shows the plasma simulation result for *N*_e,i_ = 4000. Even in this experimentally challenging regime, the measurements and the plasma simulation almost perfectly agree.

### Ultrafast dynamics

Beyond the excellent agreement with the measured kinetic distributions, these CPT simulations grant access to the dynamics of each particle. They reveal two cooling mechanisms occurring on distinct timescales: an ultrafast cooling during the plasma formation (picosecond timescale) and a subsequent process driven by the Coulomb expansion of the ionic cloud (nanosecond timescale).

**Fig. 4:**
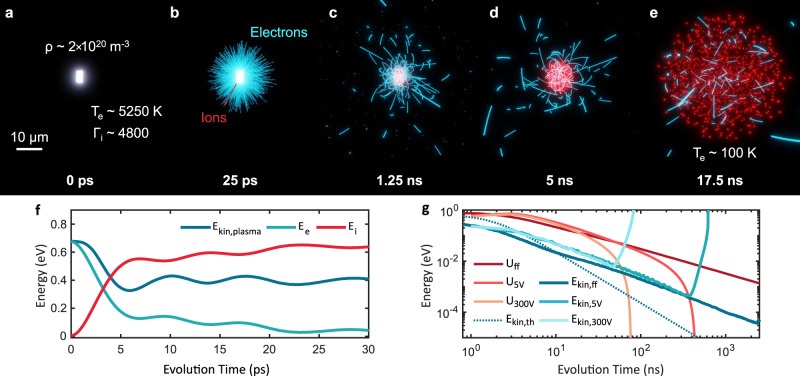
Ultrafast dynamics of a charged ultracold microplasma. **a**–**e** Snapshots from a CPT simulation tracing the electrons (blue) and ions (red) right after strong-field ionization of an ultracold atomic cloud. An initial homogeneous distribution of ions at a density of *ρ* = 2 × 10^14^ cm^−3^ and electrons at a temperature of *T*_e_ ≈ 5250 K is created in a micrometer-sized volume (**a**). The initial kinetic energy of the ions corresponds to a coupling parameter of Γ_i_ = 4800 according to Eq. (2). The electrons leave the ionization volume on a picosecond timescale and are subsequently decelerated by the core of remaining ultracold ions (**b**). While the outer electrons escape, the inner electrons are bound onto orbital trajectories in the ions’ attractive Coulomb potential forming an ultracold plasma (**c**). On a nanosecond timescale the ionic component expands driven by Coulomb repulsion and further cools the electrons to temperature below 100 K (**d**, **e**). **f** Picosecond time-evolution of the field-free plasma simulation with the total energy per particle for the electrons (light blue) and the ions (red) and the mean kinetic energy of the electrons captured within the plasma (dark blue). **g** Evolution of the kinetic energy of the trapped plasma electrons and the effective depth of the space charge potential on the nano- to microsecond timescale for field-free expansion (dark blue/red), ±*U*_ext_ = 5 V (blue/red) and ±*U*_ext_ = 300 V (light blue/red) as well as the theoretically predicted kinetic energy evolution for adiabatic electron cooling (dotted blue line).

Figure 4a–e shows snapshots of the CPT simulations for an ultracold microplasma consisting of a few thousand charged particles. While UNPs are realized at low excess energies (typically below 0.13 eV)^5^, we are able to create an ultracold plasma at high excess energies (0.68 eV), corresponding to an initial electron temperature of *T*_e_ ≈ 5250 K. Therefore, the majority of photoelectrons leaves the ionization volume within a few picoseconds, while the ions can be regarded as static (Fig. 4a).

The decrease of the electronic density in the plasma reduces the amount of shielding between the ions, which thus gain potential energy. In addition, this charge separation process gives rise to a space charge potential that strongly decelerates the escaping electrons (Fig. 4b). As a result, the kinetic energy of the electronic component is converted to potential energy of the ions. Whereas half of the electrons entirely escape the ionic core (escaping electrons), the other half is trapped within the evoked space charge potential (plasma electrons, see Supplementary Note 4 for definition). Figure 4f displays the evolution of the mean total electron (light blue line) and ion energy (red line) per particle determined by the sum of kinetic and potential energy of each component. In addition, the kinetic energy of the plasma electrons (dark blue line) is shown. Within the electronic expansion, 50% of the plasma electrons’ kinetic energy is transferred onto the potential energy of the ionic component within the first 7 ps. On this timescale, the trapped electrons are cooled down from 5250 K to about 2500 K, yielding an ultrafast cooling rate for electrons of ≈ 400 K ps^−1^. Disorder-induced heating of the electrons is negligible as the associated temperature of *T*_DIH_ ≈ 70 K in this density regime is exceeded by orders of magnitude by the initial electron temperature^9^.

The large charge imbalance of our plasma strongly influences the many-body dynamics. As depicted in Fig. 4c, the electrons are trapped in orbital trajectories within the Coulomb potential of a quasi-static ionic core. This leads to an oscillatory exchange of energy between the captured electrons and the ions (see Fig. 4f). The period of 2*π*/*ω*_p,e_ ≈ 8 ps of these oscillations is consistent with the inverse of the initial electron plasma frequency given in Eq. (1). This energy transfer between the individual electrons and the ions is predominantly in phase during the initial electron expansion but it dephases over time leading to a damping behavior (see Supplementary Note 4). In contrast to UNPs, here, the ionic plasma period always exceeds the evolution time, preventing ionic thermalization.

On a nanosecond timescale, the potential energy stored in the ions gradually translates into the kinetic energy leading to a Coulomb explosion of the plasma (Fig. 4d, e). Whereas UNPs typically exhibit hydrodynamic expansion after an equilibration due to the electrons thermal pressure^26^, in this work, the positively charged plasma expansion is dominated by the Coulomb pressure of the charge imbalance, yielding an asymptotic expansion velocity of the root-mean-square (rms) ion radius of 418 m s^−1^ (see Supplementary Note 4). This is in reasonable agreement with the expected hydrodynamic expansion velocity ${v}_{{\rm{hyd}}}=\sqrt{{k}_{\mathrm{{B}}}({T}_{\mathrm{{e},0}}+{T}_{\mathrm{{i},0}})/{m}_{\mathrm{{i}}}}=710\,{\rm{m}}\ {{\rm{s}}}^{-1}$ for tion temper/ion temperature *T*_e/i,0_^6^. The ionic expansion leads to a further reduction of the electronic temperature down to *T*_e_ ≈ 100 K within tens of nanoseconds. The simulations reveal an increasing electron coupling parameter toward Γ_e_ = 0.3 approaching significant coupling (see Supplementary Note 7).

The evolution of the mean kinetic energy of the plasma electrons as well as the depth of the effective space charge potential during the plasma expansion are shown in Fig. 4g for CPT simulations for different extraction fields (see Supplementary Note 5). In addition, the expected electron kinetic energy progression given by adiabatic cooling during the plasma expansion ${E}_{{\mathrm{kin,e}}}(t)={E}_{{\mathrm{kin,e}}}(0)(1+{t}^{2}/{\tau }_{\exp }^{2})$ is shown (dotted blue line)^6^. Here, ${\tau }_{\exp }=\sqrt{{m}_{\mathrm{{i}}}{\sigma }^{2}/[{k}_{\mathrm{{B}}}({T}_{\mathrm{{e},0}}+{T}_{\mathrm{{i},0}})]}$ denotes the plasma expansion time and *σ* is given by the initial rms ion radius. The observed electron cooling rates during the plasma expansion largely follow the prediction by the hydrodynamic model. However, the initial ultrafast electron cooling is not captured by this model.

The decrease of the ionic density over time lowers the binding Coulomb potential to the point where its gradient is exceeded by the extraction field^27^. At this point, the plasma electrons are escaping the space charge potential and are drawn to the detector. Hence, the final electron temperatures can be controlled by the extraction field, which determines the plasma lifetime and thus the duration of the electron cooling process. Without extraction field, electrons can be cooled down to sub meV energies in <1 μs.

### Plasma lifetime

**Fig. 5:**
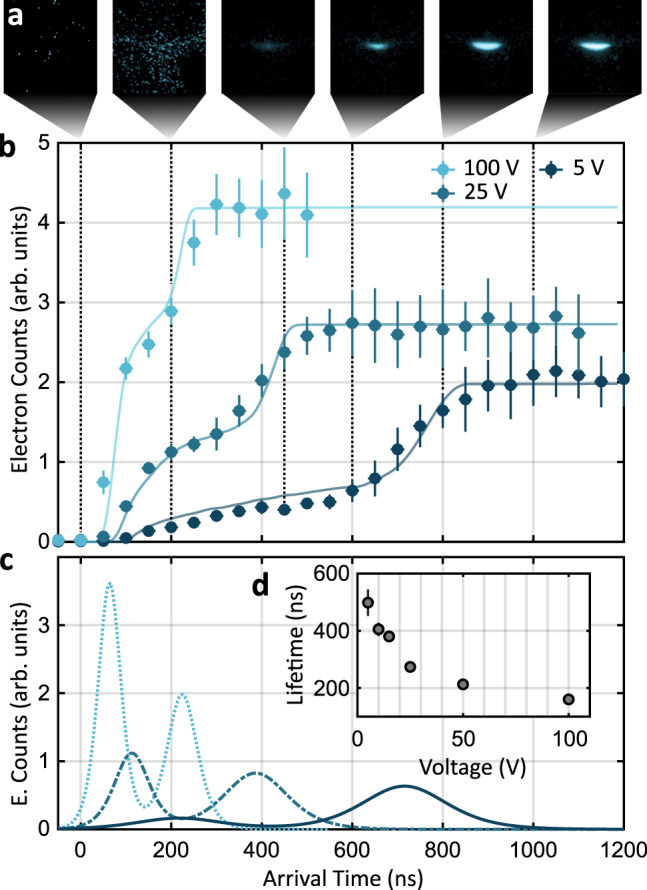
Time-resolved photoelectron detection. **a** Cumulated electron signals at an extraction field of ±*U*_ext_ = 5 V for time delays of 0, 200, 450, 600, 800, and 1000 ns after the femtosecond laser pulse (vertical dotted lines in (**b**). The signals are averaged over more than ten realizations. For a better contrast, the two first images are scaled to their maximum intensity, while the others are normalized to the maximum intensity of the last image. **b** Accumulated electron counts measured at peak intensities of 1.2 × 10^13^ W cm^−2^ for different extraction fields at ±*U*_ext_ = 100 V (light blue line), ±*U*_ext_ = 25 V (blue line) and ±*U*_ext_ = 5 V (dark blue line) as a function of the maximum arrival time. The counts are spatially integrated over the full detector area. The vertical error bars are given by the standard deviation over all realizations, while the horizontal ones indicate the time uncertainty of the repulsive voltage pulse (see Supplementary Note 9). The solid lines show the vertically scaled results of the corresponding plasma simulations. **c** Electron time-of-flight distributions obtained by the derivative of double-sigmoid fits to the data in (**b**). The two peaks represent escaping and plasma electrons. **d** Plasma lifetime for different extraction fields.

We determine the lifetime of the microplasma experimentally by implementing a gated detection scheme. In order to separate the slow plasma electrons from the fast escaping electrons, a short repulsive voltage pulse is applied onto the electron extraction electrode after a certain time *t*_delay_ to repel electrons that have not yet passed the electrode (see Supplementary Note 9). The resulting accumulated electron signals for different time delays are depicted in Fig. 5a. The escaping electrons arrive on the detector within the first 200 ns and display a homogeneous signal as expected for photoelectrons at 0.68 eV kinetic energies at these low extraction fields. Up to 600 ns, only a small fraction of cold plasma electrons is detected leaking out of the plasma during expansion. In the last 400 ns, the fraction of plasma electrons significantly increases as the expanded plasma is torn apart by the extraction field.

For a quantitative analysis, Fig. 5b shows the corresponding spatially integrated electron counts for different extraction voltages. One can clearly distinguish two plateaus that can be associated to the escaping electrons arriving first and the plasma electrons delayed by several hundreds of nanosecond^5^. The arrival time distributions obtained from the plasma simulations show an excellent agreement with respect to their temporal profile (see Fig. 5b solid lines, vertically scaled with one free parameter).

Figure 5c shows the electron arrival rates obtained from the time derivative of a double-sigmoid fit to the data in Fig. 5b (see Supplementary Note 9). The plasma lifetime corresponds to the time delay between the arrival of escaping and the plasma electrons. The measured lifetimes are plotted for the different extraction fields in Fig. 5d. For the lowest extraction field we achieve a lifetime of 498(46) ns, which is in excellent agreement with the calculated vanishing time of the space charge potential in Fig. 4g.

## Discussion

In summary, we have experimentally realized an ultracold plasma in a charge carrier density regime unexplored so far, which supports an initial ion coupling parameter of Γ_i_ = 4800. We have directly measured electron cooling from 5250 K to below 10 K within 500 ns, arising from the unique combination of a high charge carrier density and a micrometer-sized ionization volume. The CPT simulation results are in an excellent agreement with the measurements and reveal an ultrafast energy transfer of 50% of the initial electron energy onto the ionic component within the first plasma period yielding an ultrafast initial electron cooling rate of ≈ 400 K ps^−1^. The high degree of experimental control over the initial state, together with the almost perfect agreement of the CPT simulations, provides a unique model system to investigate the validity of statistical plasma models.

Besides the fundamental interest in the plasma dynamics, the low electron temperatures and enormous cooling rates may be used in the plasma-based ultracold electron sources^28^ (see Supplementary Note 11). The generated low-emittance electron bunches can be utilized for seeding the high-brilliance particle accelerators^29^ and coherent imaging of biological systems^30^. Our system links the contemporary source designs utilizing MOTs^31,32^, where the electron–cathode interactions are negligible, and traditional state-of-the-art electron sources^33–35^, where the emittance is fundamentally limited by such interactions. Our density regime indeed promotes an electron cooling mechanism based on their interaction with the ionic core acting as photoemission cathode.

By exploiting the toolbox of ultrafast dynamics further, our experimental setup allows investigating more advanced dynamical schemes. The impact of the plasma geometry can be studied by taking advantage of the nonlinearity of the strong-field ionization process in order to shape the ionization volume beyond Gaussian distributions. The interaction between several microplasmas, launched simultaneously within a BEC, can also be explored. Moreover, pump-probe schemes utilizing a second synchronized terahertz pulse for controlling the plasma evolution can offer direct experimental access to the ultrafast dynamics of the microplasma.

The photoionization of a BEC instead of a magneto-optically trapped gas paves the way toward plasmas supporting strong ion as well as electron coupling, mimicking conditions in gas planets^3^, confinement fusion^2^, or even more exotic systems like quark-gluon-plasma^7^. Indeed, strong coupling can be reached by tuning the wavelength of the femtosecond laser close to the ionization threshold, thus reducing the initial kinetic energy of the electrons by orders of magnitude. Below the ionization threshold, the large spectral bandwidth of the femtosecond laser pulses shall prevent Rydberg blockade effects and enable an efficient excitation of a dense ultracold Rydberg gas, which can form strongly coupled plasma by avalanche ionization^4,36,37^. Finally, disorder-induced heating as limiting process for Coulomb coupling, can be suppressed by loading the condensate into a 3D optical lattice that establishes an initial spatial correlation^38^.

## Methods

### ^87^Rb Bose–Einstein condensates

The ^87^Rb atoms, evaporated from dispensers, are captured in a 2D MOT used to efficiently load a 3D MOT. Bright molasses cooling allows reaching sub-Doppler temperatures (*T*_D_ = 148 μK). The atomic cloud is then loaded into a hybrid trap^39^ combining a magnetic quadrupole field and a far-detuned dipole trap at 1064 nm and further cooled by forced radio-frequency evaporation. The dipole trap beam transports the ultracold ensemble into the center of the imaging MCP detectors. After further evaporative cooling in a crossed dipole trap, quantum degeneracy can be reached.

A BEC with *N* = 4.2(3) × 10^4^ atoms is held in the far-detuned optical dipole trap at trap frequencies of *ω*_*x*,*y*_ = 2*π* × 113(3) Hz and *ω*_*z*_ = 2*π* × 128(1) Hz providing a peak atomic density of *ρ* = 2 × 10^14^ cm^−3^.

### Femtosecond laser pulses

In this work, we use laser pulses with a central wavelength of 511.4 nm and a bandwidth of 1.75 nm (FWHM). The duration of the Gaussian temporal profile is 215${\,}_{-15}^{+20}$ fs (FWHM), measured by autocorrelation. A detailed description of the pulse generation can be found in ref. ^21^.

The pulses are focused by a high-resolution microscope objective with a numerical aperture of 0.5 onto the optical dipole trap. The focal waist is measured with an additional, identical objective to *w*_1_ = 0.99(3) μm and *w*_2_ = 1.00(5) μm. The pulse energies, *E*_p_, are inferred from the measured averaged laser power, *P*, at a pulse repetition rate of 100 kHz, taking the mirror reflectivities as well as the calibrated transmission of the objective and the shielding mesh into account (see Supplementary Note 10). With the measured quantities, the applied peak intensities *I*_0_ are determined by

3 $${I}_{0}=\frac{2{P}_{0}}{\pi {w}_{1}{w}_{2}}$$

with the peak power ${P}_{0}={E}_{\mathrm{p}}/\left(\sqrt{2\pi }\tau \right)$ including the rms pulse duration $\tau ={\tau }_{{\rm{FWHM}}}/\left(2\sqrt{2{\mathrm{ln}}\,(2)}\right)$. The resulting pulse intensity distributions as well as the atomic density distribution allow determining the initial electron/ion distributions within the ionization volume (see Supplementary Note 1).

### Charged particle detection

The experimental setup enables direct detection of electrons and ions on opposite spatially resolving detectors. For this purpose, an extraction field accelerates the charged particles onto the detectors. In this work, we analyze the recorded photoelectron kinetic energy distribution.

The static extraction field is created by two opposing mesh electrodes at ±*U*_ext_, which consist of gold-plated etched copper meshes with about 70–80% permeability. After passing the meshes, the electrons are post-accelerated toward two MCPs in Chevron configuration with a channel diameter of 12 μm and a channel pitch of 15 μm attached to a P46 (Y_3_Al_5_O_12_:Ce) phosphor screen. The emitted light from the phosphor screen is recorded by a high-speed camera, which is operated at a frame rate of 1000 fps. The detection efficiency for electrons *η* ≈ 0.4 is given by the product of the transparency of the extraction meshes and the open area ratio as well as the quantum efficiency of the MCP. In order to ensure a constant, optimal quantum efficiency for all extraction voltages, the electrons are post-accelerated onto the same front potential *U*_front_ = 268 V of the MCP.

Absolute electron numbers are challenging to obtain. Indeed, when decreasing the extraction field, the absolute detection efficiency decreases as electrons with the high kinetic energies cannot be detected efficiently. Moreover, the high flux of plasma electrons on a small part of the MCP area as seen for ±*U*_ext_ = 300 V in Fig. 3c leads to electron depletion in the microchannel material and, thus, a systematic underestimation of the number of incident electrons in this part of the detector. Furthermore, detection of low kinetic energy electrons is notoriously challenging and typically limited by residual electric and magnetic fields. Therefore, it requires high experimental control over such stray fields (see Supplementary Note 3).

### CPT trajectory simulations

In order to obtain a full simulation of the experiment (including the charged particle detectors), trajectory simulations are performed using the electrostatics as well as the particle tracing module within the COMSOL Multiphysics^®^ software^40^. For this we include the 3D computer-aided design geometry of our setup into the simulation. The use of finite element methods allows calculating the electric potential landscape produced by the different electrodes (see Supplementary Note 2). In addition, a global magnetic offset field of 370 mG perpendicular to the detection axis and the ionization beam axis is included, which is used in the experiment to center the electron signal onto the detector. The trajectory simulation results in Fig. 2d are obtained for monoenergetic ensembles of electrons having randomized positions within the ionization volume and velocity directions. Due to the expansion during the time of flight, the spatial extent of the electron signal grants access to the underlying velocity distribution.

### CPT plasma simulations

The CPT simulations of the plasma dynamics are carried out with the particle tracing module within the COMSOL Multiphysics^®^ software^40^. For these simulations, *N*_e,i_ electrons and ions are randomly distributed in a cylindrical ionization volume. The particles are created monoenergetically according to the two-photon excess energies whereas the distribution of velocity directions is randomized. For *t* > 0, the 3D differential equation of motion is solved numerically for all particles including Coulomb interaction. Typical calculations for *N*_e,i_ = 4000 and few microseconds of time evolution last between 5 and 22 days while being paralleled on 35 processing units (2.2 GHz) corresponding to a CPU time of a few hundred days.

The divergence of the interparticle Coulomb potential *U*_*C*_(*r*) is circumvented by introducing a bounded interaction potential

4 $${\tilde{U}}_{{C}}(r)=\left\{\begin{array}{ll}{U}_{{C}}(r),&r\, > \, {r}_{0}\\ {U}_{{C}}({r}_{0}),&r \, \le \, {r}_{0}\end{array}\right.$$

where *r* denotes the interparticle distance and *r*_0_ = 20 nm the cutoff radius, which is chosen well below the mean initial interparticle distance. The simulations furthermore neglect interactions with the remaining neutral atoms and radiative energy losses of the charged particles.

Beyond macroscopic quantities such as the mean kinetic energies of the electron/ion ensembles, the plasma simulations offer detailed access to the dynamics at a single-particle level (see Supplementary Note 4)^41^.

## Supplementary information

Supplementary Information

## Data Availability

The data that support the findings of this study are available from the corresponding author upon reasonable request.

## Code availability

Any custom computer code used in this study is available from the corresponding author upon reasonable request.
