# Supplementary material for: Ultrafast electron cooling in an expanding ultracold plasma
Source: Nat Commun. 2021 Jan 26;12:596. doi: 10.1038/s41467-020-20815-8 (PMC7838291; doi:10.1038/s41467-020-20815-8)
Supplement: Supplementary file 1 — Supplementary Information [file 41467_2020_20815_MOESM1_ESM.pdf]

Supplementary Material: Ultrafast Electron Cooling in an Expanding Ultracold Plasma

1. IONIZATION VOLUME

In order to calculate the initial electron/ion distributions

$$\rho_{\mathrm{e/i}}(x,y,z) = \rho_{\mathrm{a}}(x,y,z) \times P(x,y,z), \quad \text{(S1)}$$

the full 3D distribution of the atomic density $\rho_{\mathrm{a}}(x,y,z)$ is modeled and multiplied with the non-linear 3D ionization probability distribution $P(x,y,z)$ given by the intensity distribution of the laser pulse. Here, z denotes the pulse propagation direction. The ionization probabilities are obtained by solving the time-dependent Schrödinger equation. We have demonstrated in a previous work that this theoretical description is in perfect agreement with the measured ionization probabilities [1].

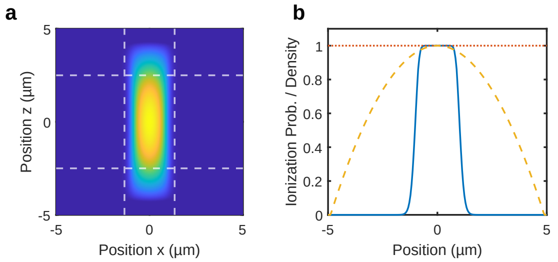

FIG. S1. **Ionization volume**. **a**. 2D projection of the simulated 3D electron/ion density distribution $\rho_{\mathrm{e/i}}(x,y,z)$ after strong-field ionization by a single pulse with $I_0 = 1.9 \times 10^{13}\ \mathrm{W\,cm^{-2}}$ The dashed white lines mark the cylindrical volume, which is used for the CPT plasma simulations. **b**. Ionization probability in x-direction (solid blue line), in z-direction (red dotted line) and normalized atomic density distribution in x-direction (dashed yellow line).

Figure S1a shows the 2D-projection of the obtained electron/ion density distribution for a pulse of $I_0 = 1.9 \times 10^{13}\ \mathrm{W\,cm^{-2}}$ together with the cylindrical volume used for the plasma simulations. Fig. S1b depicts the ionization probability in x-direction at $(y,z) = (0,0)$ (solid blue line), in z-direction at $(x,y) = (0,0)$ (red dotted line) as well as the normalized atomic density distribution in x-direction at $(y,z) = (0,0)$ (dashed yellow line) as a Thomas-Fermi profile of the BEC for the experimental trap frequencies. The atomic cloud is almost spherical as the trapping frequencies are similar in all three dimensions. Whereas the photoionization can be regarded as local in in x- and y-direction, we fully ionize the atomic ensemble in the direction of the pulse propagation z.

2. ELECTRIC FIELD CONFIGURATION

We use the Electrostatics Module within the COMSOL Multiphysics® software [2] to calculate the electrostatic field configuration for each extraction voltage. For this purpose, we include the 3D computer-aided design (CAD) geometry of our setup into the simulation. The use of finite element methods (FEM) allows for the calculation of the electric potential landscape produced by the different electrodes. We used the physics-controlled mesh option ’finer’ with a minimum/maximum element size of 0.88/12 mm.

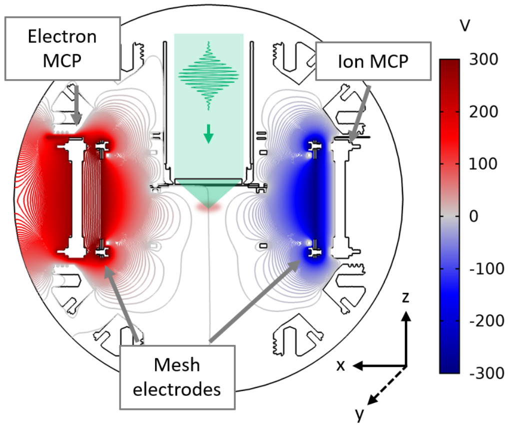

FIG. S2. **Electric extraction field.** Sectional view into the 3D-CAD model of the vacuum chamber (black lines). The electric potential obtained for $\pm U_{\mathrm{ext}} = 300$ V is depicted as equipotential lines (from blue to red). As the ion MCP and the vacuum chamber are grounded, the electric potential is dominated by the mesh electrodes and the electron MCP.

Figure S2 shows a sectional view into the vacuum chamber together with the equipotential lines obtained for $\pm U_{\mathrm{ext}} = 300$ V. Since the high-resolution objective needs to be close to the ionization volume, the reentrant window has to be shielded by a grounded high-transparency copper mesh (identical to the ones used for the electrodes) to avoid accumulation of static charge. This leads to a non-linear extraction field strongly increasing towards the detector, as well as a top-down asymmetry, which explains the non-spherical distributions observed experimentally (compare Fig. 3).

The extraction field in the interaction region can be precisely controlled by the voltages U_{ext}. However, its amplitude is not perfectly proportional to the applied voltage for low extraction fields ($\pm U_{\mathrm{ext}} = 5$ V). This is due to the voltages on the electron MCP, which give rise to an electric field on the order of 2 $\mathrm{V\,m^{-1}}$ in the center. In addition, the shielding by the vacuum chamber is included in the simulations.

3. STRAY FIELD CONTROL

The FEM simulations of the electromagnetic fields provide valuable insight into the level of control over the electric and magnetic stray fields. At an extraction voltage of $\pm U_{\mathrm{ext}} = 5$ V, which corresponds to an electric field of 4.6 $\mathrm{V\,m^{-1}}$ at the center, the extraction field still clearly dominates over the electrical stray fields. For smaller extraction fields however, our measurements deviate from the theoretical predictions due to electric fields. In our experimental setup, these electric stray fields are passively shielded by the grounded vacuum chamber and electric gradients are controlled down to the $\mathrm{V\,m^{-1}}$ level.

Helmholtz coils are used to compensate magnetic fields in all three spatial dimensions with an accuracy of 10 mG. A homogeneous compensation over the extent of the detection units is enabled by meter-sized coils. We have been working here with a magnetic field offset of 370 mG along the y-axis, which increases our energy resolution and centers the electron signal onto the detectors.

4. CPT PLASMA SIMULATIONS

The plasma simulations are based on CPT simulations and include Coulomb interaction between the charged particles (see Methods). Besides quantities such as the mean kinetic energies of the electron/ion ensembles, the CPT plasma simulations provide detailed access to the dynamics of each charged particle. Figure S3a shows the time-evolution of the distance from the ionization center of single plasma electrons (blue lines) for the simulation depicted in Fig. 4f-g without extraction field. For clarity, the graph only depicts a random selection of 31 plasma electrons. One clearly identifies an oscillatory motion for the different particles exhibiting different frequencies ranging from hundreds of gigahertz to a few megahertz. The frequency is decreasing with increasing amplitude of the oscillations due to screening by the more closely bound electrons. The maximal ion radius (dashed red line) given by the maximum of all ion distances from the ionization center is used to distinguish between plasma and escaping electrons. In this simulation electrons are regarded as plasma electrons, if their distance at t = 2500 ns is less than twice the maximum ion radius.

In addition, the rms electron/ion radius is given for each time step (bold solid blue line / solid light red line). The asymptotic expansion velocity of the rms ion radius of $v_{\mathrm{i,rms}} = 418\ \mathrm{m\,s^{-1}}$ is in reasonable agreement with the expected plasma expansion velocity $v_{\mathrm{hyd}} = \sqrt{k_{\mathrm{B}}\left(T_{\mathrm{e},0} + T_{\mathrm{i},0}\right)/m_{\mathrm{i}}} \approx 710\ \mathrm{m\,s^{-1}}$ for hydrodynamic expansion (dark red dotted line) at an initial electron temperature of $T_{\mathrm{e},0} = 5250$ K and negligible initial ion temperature $T_{\mathrm{i},0}$ [3].

Figure S3b depicts the corresponding kinetic energies of the plasma electrons for the first 100 ps (blue lines) as well as the mean kinetic energy of all 1961 plasma

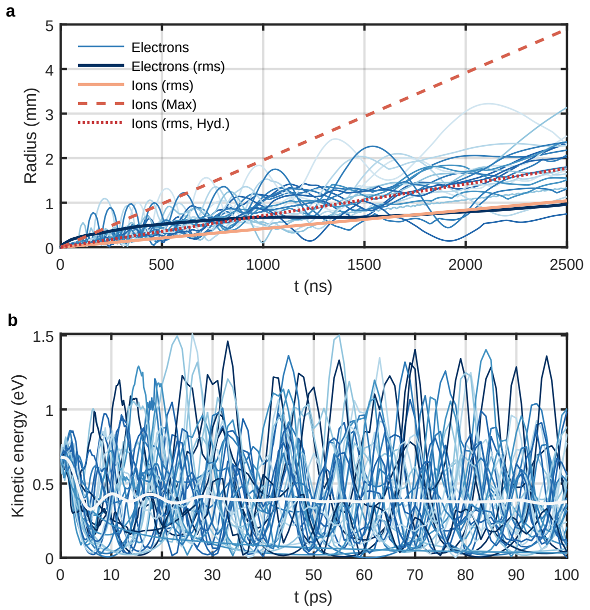

FIG. S3. **Plasma dynamics at the single particle level.** The dynamics is shown for a generic selection of 31 plasma electrons out of $N_{\mathrm{e/i}} = 4000$ simulated particles. **a**. Time-evolution of the distance to the ionization center for the individual plasma electrons (blue lines) in the field-free plasma simulation (see Fig. 4f-g) together with the rms radius of the electron/ion ensemble (bold solid blue line / solid light red line) as well as the maximal ion radius (dashed red line). In addition, the rms ion radius evolution expected from hydrodynamic expansion is given (dark red dotted line). **b**. Time-evolution of the single electron kinetic energies (blue lines). The mean kinetic energy of all plasma electrons is given by the bold white line.

electrons (bold white line). The observed oscillations in the mean kinetic energy are caused by the oscillatory motion of the electrons with similar frequencies and the apparent damping is evoked by their dephasing.

For the simulations including an extraction field, the differentiation between plasma and escaping electrons is more challenging, since electrons enter large orbits, where the non-linear extraction field become dominant, and escape from the plasma. Thus, the extraction fields reduce the number of plasma electrons by about 80% over time. For the simulations shown in Fig. 4g at $\pm U_{\mathrm{ext}} = 300$ V and $\pm U_{\mathrm{ext}} = 5$ V electrons are regarded as plasma electrons, if their distance from the ionization center after 40 ns / 300 ns is less than 1 mm.

5. EFFECTIVE SPACE CHARGE POTENTIAL

The CPT plasma simulations furthermore provide access to the space charge potential well created by the unpaired ions during the plasma expansion. The extraction field E_{ext} adds up to the 1D space charge potential

along the detection axis lowering the effective trapping potential

$$U_{\text{eff}}(r) = U(r) - E_{\text{ext}} \cdot r, \tag{S2}$$

where r denotes the distance to the ionization center in the direction of the extraction field. Figure 4g (red lines) shows the evolution of the effective space charge potential depth for the plasma simulations without extraction field as well as for extraction voltages of $\pm U_{\text{ext}} = 5$ V and $\pm U_{\text{ext}} = 300$ V (corresponding to $E_{\text{ext}} = 4.6\ \text{V m}^{-1}$ and $E_{\text{ext}} = 162\ \text{V m}^{-1}$). Here, for each time-step $U(r)$ is approximated by the Coulomb potential of a homogeneously charged sphere

$$U(r) = \begin{cases} \frac{Q}{8\pi\epsilon_0 R}\left(3 - \frac{r^2}{R^2}\right), & r \leq R \\ \frac{Q}{4\pi\epsilon_0 r}, & r > R \end{cases} \tag{S3}$$

where the radius R is given by the maximal ion radius and the charge $Q = e\cdot N_{\text{diff}}$ is given by the difference N_{diff} of the number of ions and electrons within the maximum ion radius. The potential depth is determined by the difference of the local maximum $U_{\text{eff}}(r_{\text{max}})$ and the local minimum $U_{\text{eff}}(r_{\text{min}})$ of the effective potential at

$$r_{\text{min}} = \frac{4\pi\epsilon_0 E_{\text{ext}} R^3}{Q} \text{ and } r_{\text{max}} = \sqrt{\frac{Q}{4\pi\epsilon_0 E_{\text{ext}}}}. \tag{S4}$$

6. PLASMA FORMATION

For plasma formation, the depth of the space charge potential has to exceed the electronic excess energy $E_{\text{kin,e}}$. Thus, for a given excess energy and a Gaussian spatial distribution of charge carriers, the creation of a minimum ion number $N^* = E_{\text{kin,e}}/U_0$ is required, where $U_0 = \sqrt{\frac{2}{\pi}} \frac{e^2}{4\pi\epsilon_0\sigma}$ and σ denotes the rms radius of the ionic distribution [4]. The experimentally realized ionic distribution is approximated by a Gaussian distribution with the arithmetic mean radius of $\sigma = (2\times1.35\ \mu\text{m}+5\ \mu\text{m})/3$ leading to a critical ion number of $N^* = 960$.

Figure S4 shows the measured brightness at $\pm U_{\text{ext}} = 300$ V in an elliptical area around the plasma electrons on the detector. The number of plasma electrons, as signature of the plasma formation, displays a strong dependency on the critical charge carrier density. This density has been varied either by the pulse intensity (from $0.1 \times 10^{13}\ \text{W cm}^{-2}$ to $1.7 \times 10^{13}\ \text{W cm}^{-2}$) or the atomic density (from $6 \times 10^{17}\ \text{m}^{-3}$ to $\rho = 1.3 \times 10^{20}\ \text{m}^{-3}$).

In order to vary the atomic density, we modify the evaporation efficiency, which leads to a reduced number of atoms in the final optical dipole trap. The density is scanned from an ultracold thermal cloud with $\rho = 6\times10^{17}$ m^{-3} to an almost pure condensate with $\rho = 1.3 \times 10^{20}$ m^{-3}. The densities are determined by the analysis of the optical density distributions in a time-of-flight measurement recorded by absorption imaging for different timesteps after switching off the optical dipole trap.

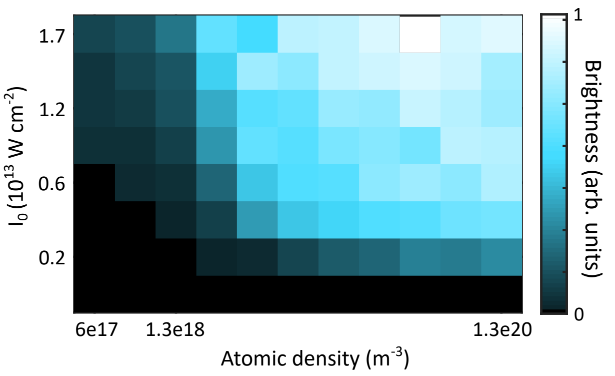

FIG. S4. **Critical charge carrier density for plasma formation.** Measured brightness of the electron signal in an elliptical area enclosing low kinetic energy electrons. The atomic target density has been varied over three orders of magnitude and the pulse intensity from $0.1 \times 10^{13}\ \text{W cm}^{-2}$ to $1.7 \times 10^{13}\ \text{W cm}^{-2}$.

Whereas the calculation of in-situ atomic densities is reliable for the limiting cases of a fully condensed or thermal atomic sample, it is known to be difficult for partly condensed samples as the determination requires a bimodal fit using a Gaussian as well as the Thomas-Fermi density model. However, the critical number of ionized atoms can be extracted from the numbers of detected electrons for each intensity density combination. As the ionization volume slightly increases with increasing peak intensity, the critical number increases as well. While for $I_0 = 1.7 \times 10^{12}\ \text{W cm}^{-2}$ around 500 ions need to be created, for $I_0 = 1.9 \times 10^{13}\ \text{W cm}^{-2}$ approximately 1000 electrons are required, which agrees well with the expected value of $N^* = 960$.

7. PLASMA PARAMETERS

During the expansion, the densities and kinetic energies of the plasma vary over several orders of magnitude. Figure S5 displays the time evolution of central parameters extracted from the CPT plasma simulation. Figure S5a shows the evolution of the electron and ion density in the center of the plasma. The electron density drops within the initial expansion and stays constant for the first nanosecond. As the ionic component expands, the electron and ion density both decrease and even fall below typical initial UNP densities. The density evolution determines the plasma frequency as well as the plasma period given in Fig. S5b. The decrease of the electron density explains the deceleration of the electronic orbital oscillations during the plasma expansion. In addition, the ionic plasma frequency significantly decreases within the first plasma period (given by the initial

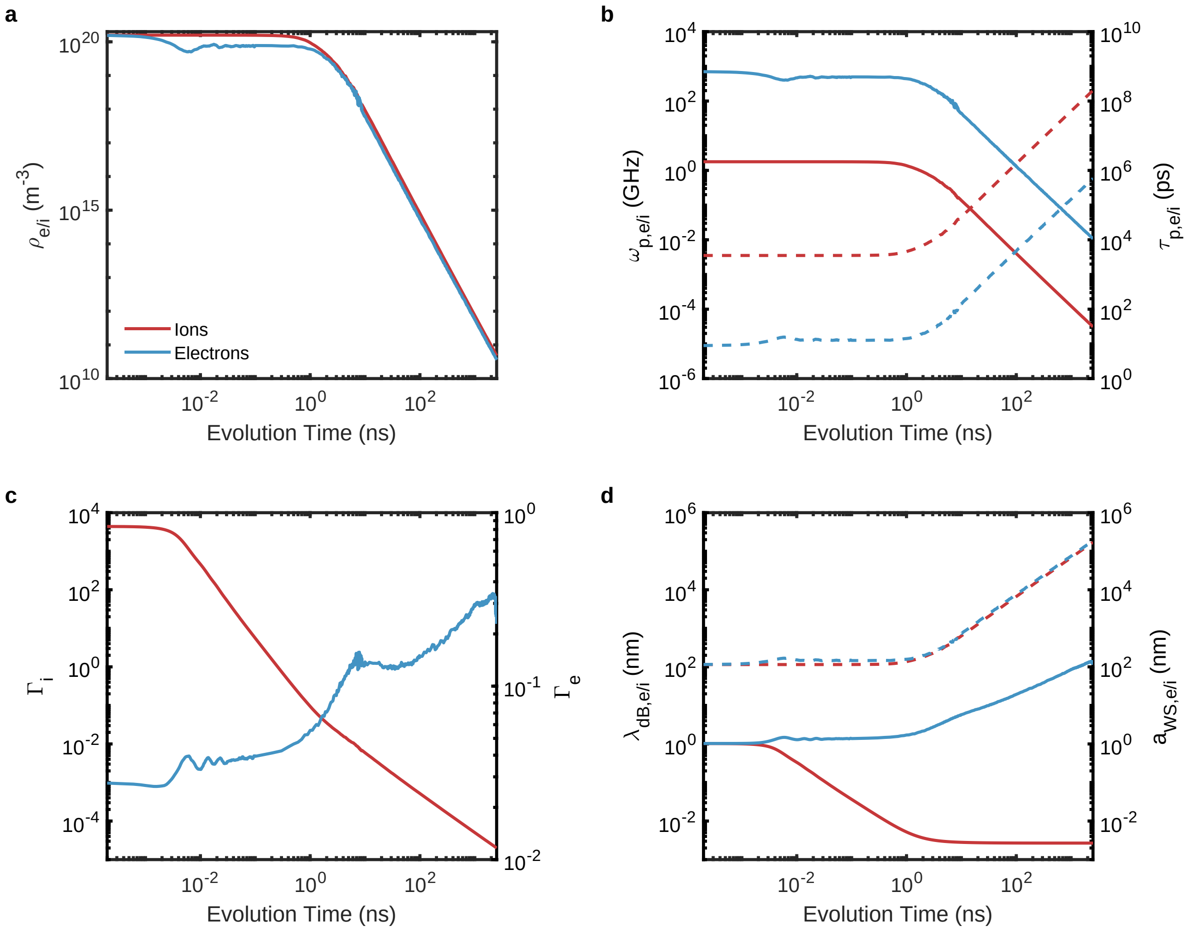

FIG. S5. **Time-evolution of the simulated plasma parameters. a.** Density of electrons (blue line) and ions (red line) in a spherical volume around the ionization center. **b.** Electron/ion plasma period $\tau_{\mathrm{p,e/i}} = 2\pi\omega^{-1}_{\mathrm{p,e/i}}$ (dashed blue/red line) and frequencies $\omega_{\mathrm{p,e/i}}$ (solid blue/red lines). **c.** Coulomb coupling parameter $\Gamma_{\mathrm{e,i}}$ of the electron/ion component (blue/red line) determined by the particle densities and the plasma electron/ion mean kinetic energy. **d.** Electron/ion de Broglie wavelength $\lambda_{\mathrm{dB,e/i}}$ (solid blue/red line) and Wigner-Seitz radius $a_{\mathrm{WS,e/i}}$ (dashed blue/red line).

ionic density). In contrast to UNP, where plasma expansion can be regarded as slow in relation to the inverse ionic plasma frequency, here, the ionic plasma period exceeds the plasma expansion duration, thus preventing ionic thermalization.

In Fig. S5c the ion and electron coupling parameters are depicted. The ionic coupling parameter decreases after the first electron plasma period when charge imbalance is established due to ionic acceleration and increasing interparticle distance. On the contrary, the electronic coupling parameter increases during the plasma expansion since the electron temperature decreases over orders of magnitude. The simulations reveal a maximum coupling parameter of $\Gamma_{\mathrm{e}} = 0.3$ approaching significant electron coupling.

Electron temperatures in the Kelvin domain raise the question of quantum degeneracy for the electronic ensemble. Fig. S5d illustrates the electron/ion de Broglie wavelengths $\lambda_{\mathrm{dB,e/i}}$ at different expansion times. Whereas the ionic wavelength quickly decreases, the electrons reach a maximum de Broglie wavelength on the order of $\lambda_{\mathrm{dB,e}} \approx 100$ nm at the end of the plasma expansion. However, the ratio of mean interparticle distance given by the Wigner-Seitz radius $a_{\mathrm{WS,e/i}}$ (dashed blue/red line) and the de Broglie wavelength never exceeds 1.3 %, which yields $E_{\mathrm{kin,e}}/E_{\mathrm{F}} > 6000$, where $E_{\mathrm{F}} = \frac{\hbar^2}{2m_{\mathrm{e}}}\left(3\pi^2\rho_{\mathrm{e}}\right)^{2/3}$ denotes the electron Fermi energy with the reduced Planck constant $\hbar$. Thus, a quantum mechanical description required for a fermionic ensemble close to degeneracy can be safely disregarded.

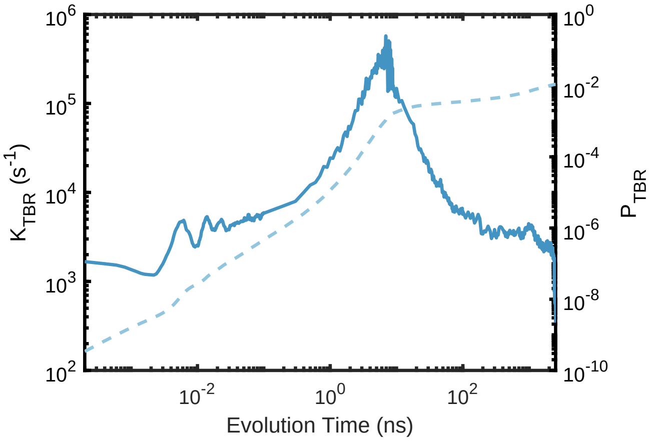

FIG. S6. **Three-body recombination in the ultracold microplasma.** Time-evolution of the TBR rate K_{TBR} per ion during the plasma expansion (solid blue line) as well as the resulting time-integrated recombination probability P_{TBR} per ion (dashed blue line).

8. THREE-BODY RECOMBINATION

For ultracold plasma in the density and temperature regime described in this manuscript, three-body recombination (TBR) is expected to be the dominant process of electron-ion recombination. The TBR rate K_{TBR} per ion according to classical TBR theory is given by $K_{\mathrm{TBR}} \approx 3.8 \times 10^{-9}\ T_{\mathrm{e}}^{-9/2} \rho_{\mathrm{e}}\ \mathrm{s}^{-1}$, where T_{e} is the electron temperature in K and the electron density ρ_{e} is given in cm^{-3} [3]. Figure S6 shows the calculated TBR rate per ion (solid line) as well as the time-integrated TBR probability per ion (dashed line). After 2.5 µs of plasma expansion, a cumulated TBR probability of approximately 1% is reached. As a result, the plasma lifetime is expected to be on the order of 100 µs before a significant fraction of Rydberg excitations are created. However, on the ten microsecond timescale, when the plasma is dilute and collisions barely occur, radiative and dielectronic recombination might further limit the plasma lifetime.

9. TIME-RESOLVED ELECTRON DETECTION

The experimental setup gives access to the distribution of arrival times of the detected electrons by a gated detection scheme (Fig. S7). For this purpose, a repulsive voltage pulse is applied to the electron extraction mesh after a variable delay t_{delay} after the femtosecond laser pulse. The rapidly switched potential prevents electrons from passing the extraction mesh for time-of-flight durations $\tau_{\mathrm{ToF}} > t_{\mathrm{delay}}$. The applied pulse has a duration of 2 µs and is capacitively coupled onto the meshes. This enables to measure the accumulated electron signal up to t_{delay}, while maintaining the spatial resolution. The estimated timing uncertainty of 30 ns is caused by the temporal jitter and the pulse rise time.

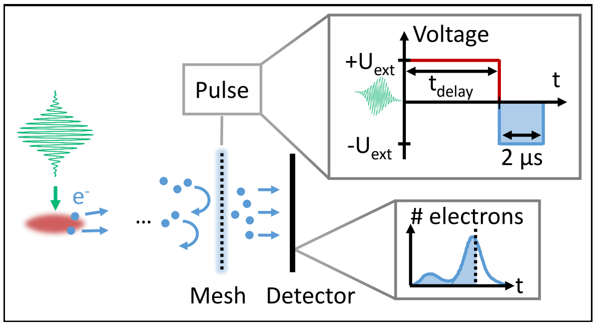

FIG. S7. **Photoelectron time-of-flight measurement.** The photoelectrons created in the BEC are accelerated towards the detector by a variable extraction field. After a variable time t_{delay}, a microsecond voltage pulse switches the polarity of the extraction field. The resulting repulsive electric field prevents electrons with arrival times $t_{\mathrm{ToF}} > t_{\mathrm{delay}}$ from passing the mesh. Thus, the detector records the time-integrated electron signal up to t_{delay}.

In order to analyze the obtained temporal profiles (see Fig. 5b) quantitatively, a double-sigmoid function

$$f(t) = \frac{a_1}{1 + e^{-b_1 \cdot (t - c_1)}} + \frac{a_2}{1 + e^{-b_2 \cdot (t - c_2)}} \qquad \text{(S5)}$$

is fitted to the measured data. Figure S8 shows the fit functions for $\pm U_{\mathrm{ext}} = 100$ V, $\pm U_{\mathrm{ext}} = 50$ V, $\pm U_{\mathrm{ext}} = 25$ V, $\pm U_{\mathrm{ext}} = 15$ V, $\pm U_{\mathrm{ext}} = 10$ V and $\pm U_{\mathrm{ext}} = 5$ V (solid lines, from light blue to dark blue). The two inflection points for the double-sigmoid functions are given by c_1 and c_2 (vertical dashed lines). The spectra in Fig. 5c are the time derivatives of the fitted functions. The arrival time difference $c_2 - c_1$ determines the plasma lifetime shown in Fig. 5d. The error bars are given by the 95% confidence interval for the inflection points.

10. INTENSITY CALIBRATION

The experimental setup only provides access to the femtosecond laser power before passing the high resolution microscope objective. As the transmittance α_{T} critically depends on pointing and angle of the incident laser beam, the actual peak intensities inside the vacuum chamber have to be calibrated. The averaged laser power used for the calculation of the applied peak intensity (see Methods) is given by $P = \alpha_{\mathrm{T}} P_{\mathrm{front}}$. Here, P_{front} denotes the power in front of the objective, which is measured through a circular aperture with the same diameter as the objective aperture (4 mm) at a pulse repetition rate of 100 kHz. Figure S9 shows the measured number of electrons for different powers P_{front} at an extraction voltage of $\pm U_{\mathrm{ext}} = 200$ V (data points). The solid lines depict the expected numbers of electrons assuming a transmittance of 0.2, 0.1 and 0.05, which are calculated by use of the absolute ionization probabilities reported in [1] as

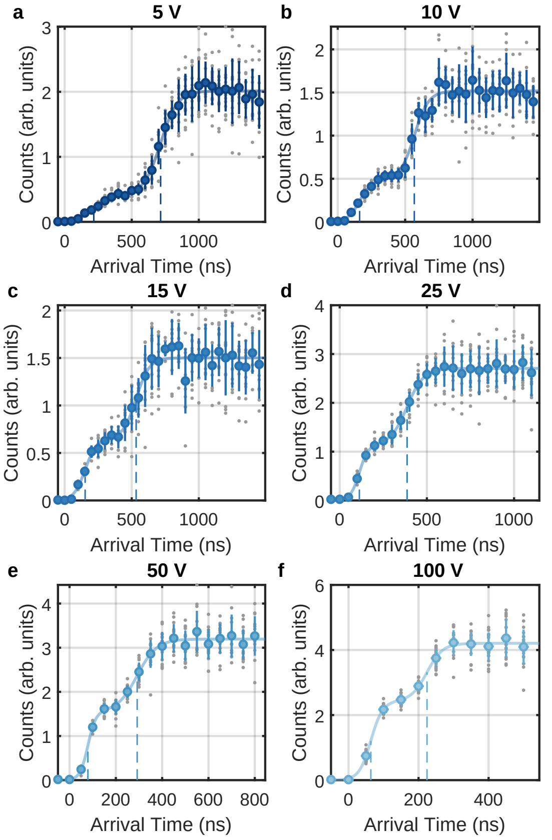

FIG. S8. **Electron arrival time. a.-f.** Accumulated electron counts measured at a peak intensity of 1.2×10^{13} W cm^{-2} for different extraction voltages (see Fig. 5b) (black data points). The vertical error bars of the binned data (blue data points) are given by the standard deviation over all realizations and the horizontal ones indicate the time uncertainty of the repulsive voltage pulse. The solid lines show the double-sigmoid functions according to Eq. (S5) obtained by a fit to the data points. The dashed lines depict the inflection points c_1 and c_2 of each sigmoid function.

well as the beam waist measured with an identical objective (see Methods). The best agreement is obtained for $\alpha_\mathrm{T} = 0.1$.

11. ULTRACOLD ELECTRON SOURCE

The electron cooling mechanisms in ultracold plasma can be exploited for plasma-based ultracold electron sources [5] producing low-emittance electron bunches. These bunches can be used to seed high-brilliance particle accelerators [6] and for coherent imaging of biological systems [7]. With the final electron temperature of $T_\mathrm{e} \approx 10$ K and an rms electron bunch radius σ_r= 0.52 mm, we achieve a normalized rms emittance of

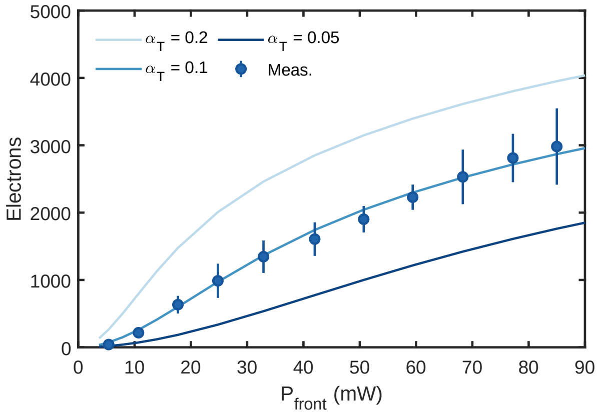

FIG. S9. **Intensity Calibration**. Measured numbers of electrons for different laser powers P_front at $\pm U_\mathrm{ext} = 200$ V (data points) as well as the calculated numbers of electrons assuming a transmittance of 0.2, 0.1 and 0.05 (solid lines). The vertical error bars are given by the standard deviation over all realizations.

$\epsilon_\mathrm{r} = \sigma_\mathrm{r} \cdot \sqrt{k_\mathrm{B} T_\mathrm{e}/m_\mathrm{e} c^2} = 21$ nm rad and a relative transverse coherence length $C_\perp = \hbar/(m_\mathrm{e} c \epsilon_\mathrm{r}) = 2 \times 10^{-5}$. In our experimental setup, the electron excess energy can be reduced further by working closer to the ionization threshold, allowing to approach the value of $C_\perp = 10^{-3}$ required for single-shot electron diffraction [8].

[1] Wessels, P. *et al.* Absolute strong-field ionization probabilities of ultracold rubidium atoms. *Commun. Phys.* **1**, 32 (2018).

[2] COMSOL Multiphysics® v. 5.4. www.comsol.com. COMSOL AB, Stockholm, Sweden.

[3] Killian, T., Pattard, T., Pohl, T. & Rost, J. Ultracold neutral plasmas. *Phys. Rep.* **449**, 77–130 (2007).

[4] Killian, T. C. *et al.* Creation of an Ultracold Neutral Plasma. *Phys. Rev. Lett.* **83**, 4776–4779 (1999).

[5] Claessens, B. J., van der Geer, S. B., Taban, G., Vredenbregt, E. J. D. & Luiten, O. J. Ultracold Electron Source. *Phys. Rev. Lett.* **95**, 164801 (2005).

[6] McCulloch, A. J. *et al.* Arbitrarily shaped high-coherence electron bunches from cold atoms. *Nat. Phys.* **7**, 785–788 (2011).

[7] Speirs, R. W. *et al.* Single-shot electron diffraction using a cold atom electron source. *J. Phys. B: At., Mol. Opt. Phys.* **48**, 214002 (2015).

[8] Engelen, W. J., van der Heijden, M. A., Bakker, D. J., Vredenbregt, E. J. D. & Luiten, O. J. High-coherence electron bunches produced by femtosecond photoionization. *Nat. Commun.* **4**, 1693 (2013).
